# A Multilayer Perceptron Neural Network Model to Classify Hypertension in Adolescents Using Anthropometric Measurements: A Cross-Sectional Study in Sarawak, Malaysia

**DOI:** 10.1155/2021/2794888

**Published:** 2021-12-07

**Authors:** Soo See Chai, Whye Lian Cheah, Kok Luong Goh, Yee Hui Robin Chang, Kwan Yong Sim, Kim On Chin

**Affiliations:** ^1^Faculty of Computer Science and Information Technology, University of Malaysia Sarawak (UNIMAS), Malaysia; ^2^Department of Community Medicine and Public Health, Faculty of Medicine and Health Sciences, University of Malaysia Sarawak (UNIMAS), Malaysia; ^3^School of Science and Technology, International University College of Advanced Technology Sarawak (i-CATS University College), Malaysia; ^4^Faculty of Applied Sciences, Universiti Teknologi MARA, Cawangan Sarawak, 94300 Kota Samarahan, Sarawak, Malaysia; ^5^School of Engineering, Faculty of Engineering, Computing and Science, Swinburne University of Technology Sarawak Campus, Malaysia; ^6^Faculty Computing and Informatics, Universiti Malaysia Sabah (UMS), Malaysia

## Abstract

This study outlines and developed a multilayer perceptron (MLP) neural network model for adolescent hypertension classification focusing on the use of simple anthropometric and sociodemographic data collected from a cross-sectional research study in Sarawak, Malaysia. Among the 2,461 data collected, 741 were hypertensive (30.1%) and 1720 were normal (69.9%). During the data gathering process, eleven anthropometric measurements and sociodemographic data were collected. The variable selection procedure in the methodology proposed selected five parameters: weight, weight-to-height ratio (WHtR), age, sex, and ethnicity, as the input of the network model. The developed MLP model with a single hidden layer of 50 hidden neurons managed to achieve a sensitivity of 0.41, specificity of 0.91, precision of 0.65, *F*-score of 0.50, accuracy of 0.76, and Area Under the Receiver Operating Characteristic (ROC) Curve (AUC) of 0.75 using the imbalanced data set. Analyzing the performance metrics obtained from the training, validation and testing data sets show that the developed network model is well-generalized. Using Bayes' Theorem, an adolescent classified as hypertensive using this created model has a 66.2% likelihood of having hypertension in the Sarawak adolescent population, which has a hypertension prevalence of 30.1%. When the prevalence of hypertension in the Sarawak population was increased to 50%, the developed model could predict an adolescent having hypertension with an 82.0% chance, whereas when the prevalence of hypertension was reduced to 10%, the developed model could only predict true positive hypertension with a 33.6% chance. With the sensitivity of the model increasing to 65% and 90% while retaining a specificity of 91%, the true positivity of an adolescent being hypertension would be 75.7% and 81.2%, respectively, according to Bayes' Theorem. The findings show that simple anthropometric measurements paired with sociodemographic data are feasible to be used to classify hypertension in adolescents using the developed MLP model in Sarawak adolescent population with modest hypertension prevalence. However, a model with higher sensitivity and specificity is required for better positive hypertension predictive value when the prevalence is low. We conclude that the developed classification model could serve as a quick and easy preliminary warning tool for screening high-risk adolescents of developing hypertension.

## 1. Introduction

The mortality rate of heart and blood vessel disease is increasing globally. Among the diverse risk factors, hypertension turns out to be the most contributing element for this specific noncommunicable disease, particularly for premature cardiovascular disease [1]. Coronary heart disease, stroke, heart failure, dementia, aneurysm, and renal failure are some consequences that are closely linked to hypertension [2, 3]. In addition, hypertension was found to raise the severity and mortality rate of COVID-19 by around 2.5 times especially in elderly patients who are more than 60 years old [4].

Hypertension is characterized as blood pressure ≥ 140 mm Hg systolic and/or ≥90 mm Hg diastolic for adults, and its prevalence has become a worldwide health burden. In adolescents, hypertension is interpreted as blood pressure of ≥130 mm Hg systolic and/or ≥80 mm Hg diastolic [5]. Due to the global widespread of obesity and physical inactivity in children and adolescents, hypertension in this group has become an increasing health problem, yet often overlooked [6]. It was discovered that the risk factor levels of cardiovascular disease from children and adolescents persist into adulthood, which in turn increases the probability of heart and blood vessel disease events later in life [7]. Therefore, the prediction of adolescents at risk of hypertension before adulthood is crucial to implement better prevention and control programs [8]. Furthermore, childhood and adolescence are the crucial stages for hypertension control and prevention prior to any further clinical symptoms related to hypertension-associated cardiovascular disease [9]. The prevalence of hypertension was reported to be 24.5% among adolescents in Malaysia in a recent study [10].

Anthropometric indices are gradually trusted by scientists to be the mandatory factors in identifying the risk of heart disease [11]. The use of anthropometric indices promises a simple, inexpensive, efficient, and reliable initial screening technique for hypertension [12]. Many anthropometric indices are used to define obesity-associated hypertension. These include the most commonly used body mass index (BMI), waist circumference (WC), weight-to-hip ratio (WHR), and weight-to-height ratio (WHtR) [13]. Nonetheless, research shows that the predictive powers of anthropometric measures for hypertension are countries and ethnicities dependent [14].

The emergence of machine learning (ML) in the medical field has revealed the insight of new techniques for hypertension prediction. ML techniques could be used as an early prediction for hypertension disease and could serve as a supporting tool or second opinion in assisting medical doctors in making timely decisions [15]. Artificial neural network (ANN) models have shown to be a powerful ML technique and exhibited great success in disease prediction and classification [16]. Although the ANN has been extensively used to investigate risk factors for hypertension, the utilization of anthropometric, demographic, and lifestyle indices as the estimator for hypertension prediction did not outperform prediction models that use biomedical estimators. Furthermore, current research work using ML did not report how meaningful or clinically useful a classifier might be when looking at the prevalence of hypertension for a population. Therefore, there is a need to bridge this research gap by understanding whether the use of simple anthropometric is feasible for hypertension prediction and how clinically beneficial the developed model is.

In two earlier works [17, 18], the prevalence of hypertension in Sarawak adolescents and its relationships with anthropometric indices were analyzed using multivariate logistic regression and the stepwise logistic regression statistical approach. This research work is an evolution of the previous two studies by focusing on the use of an artificial neural network model. The purpose of this research is fourfold: (a) investigate which anthropometric indices are important for adolescents hypertension prediction, (b) develop an artificial neural network model for hypertension prediction focusing on the use of anthropometric indices based on a cross-sectional research work conducted in Sarawak, Malaysia, (c) analyze whether hypertension in adolescents could be reliably predicted using anthropometric indices, and (d) assess how clinically beneficial the developed model is.

## 2. Related Work

Many researchers have implemented ANN models for hypertension prediction, and some of these recent researches are [19–30]. Among these, Bani-Salameh et al. [26] developed a multilayer perceptron (MLP) neural network model with six inputs: age, weight, fat ratio, blood pressure, alcohol, and smoking; one hidden layer and one output layer of hypertension and nonhypertension classes were implemented to train and test a sample size of 760 patients. They managed to achieve a correct classification rate of 68.7% with a measured Area Under the Receiver Operating Characteristic (ROC) curve (AUC) of 0.618. In addition, the authors compared the classification results of the MLP model with the *k*-nearest neighbour (KNN) and Support Vector Machine (SVM) and concluded that MLP outperformed these two models. The analysis on the independent variables revealed that blood pressure was the most important variable while smoking was the least significant variable.

In another study by López-Martínez et al. [27], a three-layered ANN model with rectified linear activation function (ReLU) in the hidden layers to classify hypertension and nonhypertension patients using sex, race, body mass index (BMI), kidney disease, and diabetes as the input features was implemented. A large imbalance sample size of 24,434 with 60.71% nonhypertensive and 30.29% hypertensive patients was used. The ANN model implemented with seven inputs, 3 hidden neuron layers with 64, 32, and 16 nodes, respectively, and 2 outputs managed to produce classification results with a sensitivity of 40%, specificity of 87%, precision of 57.8%, and AUC of 0.77. In their earlier work [28], a logistic regression model was used on the data set from the same source but smaller size (19,709), and they achieved classification results with a sensitivity of 77%, specificity of 68%, precision of 32%, and AUC of 73% (95% CI [0.70–0.76]). Although the total number of samples used was slightly smaller in [28] as compared to their work in [27], it showed that the use of the ANN model could produce a better classification result.

A gradient descent backpropagation neural network model with four hidden units and 0 momentum value produced the best AUC (0.67), specificity (88%), sensitivity (30.6%), and precision (57.43%) results in the research work by Sakr et al. [29]. The features used included age, metabolic equivalents (METS), resting systolic blood pressure, peak diastolic blood pressure, resting diastolic blood pressure, coronary artery disease, the reason for the test, history of diabetes, percentage of heart rate achieved, race, history of hyperlipidemia, aspirin use, and hypertension response. The total number of patients was 23,095 with ages ranged between 17 and 96.

A study focusing on predicting the systolic and diastolic blood pressure of archers aged between 13 and 20 was carried out using an ANN model in [30] using a small sample size of 50 targets. The ANN model used only the calf circumference as the input variable. They reported the results for systolic and diastolic blood pressure prediction in terms of *R*^2^ (0.95, 0.95), mean absolute percentage error (MAPE) (0.05, 0.06), means of mean absolute error (MAE) (6.55, 4.44), and root mean square error (RMSE) (78.05, 35.51).

There are other earlier studies [31–35] that utilized the ANN for hypertension classification, and each of these research works exhibited their cost and values. From the most recent and relevant research mentioned above, it could be concluded that the use of anthropometric indices together with sociodemographic and lifestyle parameters is beneficial as an initial screening for hypertension. As self-reported diabetes and hypertension are not reliable [36] and the lifestyle parameters reporting are subjective [37], in our work, only simple anthropometric measurements together with sociodemographic data are used as the features to predict cases of hypertension. We want to look into how basic anthropometric measurements combined with sociodemographic data may be used to predict hypertension in adolescents and which variables contribute to predicting hypertension. The classification results derived from this study would reveal whether hypertension in adolescents could be predicted accurately using the anthropometric indices. Several performance assessment measures, such as ROC, AUC, sensitivity, specificity, accuracy, RMSE, MAE, and MAPE, were provided as a way to benchmark the constructed models in the aforementioned review. However, the question of whether the developed model is significant, particularly in terms of clinical utility in a population with a given hypertension prevalence, remains unanswered.

## 3. Method

### 3.1. Data Source and Study Population

A cross-sectional study assessing the blood pressure of secondary school children aged between 13 and 17 years in Sarawak was carried out for 7 months from 9 March 2016 to 27 September 2016. Ethical approval was obtained from the Medical and Ethical Committee of Universiti Malaysia Sarawak (UNIMAS/TNC (AA)-03.02/06-11 Jld.3(1)) and the Ministry of Education Malaysia.

Sarawak is the largest state in Malaysia located on the island of Borneo. According to the Department of Statistics Malaysia [38], in the year 2019, the population in Sarawak is estimated to be 2.81 million with more than 40 subethnic groups. Each of these subethnic groups has its own language, lifestyle, and culture [17]. Iban, Chinese, Malay, Bidayuh, Melanau, and Orang Ulu are among the six major subethnics in Sarawak.

A total of 19 schools participated in this study with 14 of these schools classified as rural while the other 5 schools were classified as urban. For each school, a class was randomly chosen from each of the schooling levels of secondary one to secondary six. Only participants without physical and mental disability, no prediagnosed hypertension, and sickness that might lead to secondary hypertension were enrolled in the study. Data collection was carried out by a team of trained laboratory personnel. According to the Ministry of Education, the total number of students aged 13 to 17 in Sarawak in February 2014 was 200,130. Equation (1) is used to compute the required sample size (*s*) for a finite group [39]:

(1) $$\begin{array}{c} s=\frac{X^{2}NP\left(1-P\right)}{d^{2}\left(N-1\right)}+X^{2}P\left(1-P\right), \end{array}$$

where *X* is the *z*-score for 99% confidence interval (2.58), *N* is the population size (200130), *P* is the population proportion (assume to be 0.5 as this would produce the maximum sample size), and *d* is the degree of accuracy or margin of error (0.028).

According to the calculations, a sample size of 2124 was required.

Sociodemographic information comprising the age, sex, and ethnicity of each participant was recorded. Next, the trained personnel would gather the anthropometric data from the participants. The anthropometric data collection was done using a SECA body meter and portable weighing scale. During weighing, the participants were asked to take off their footwear. In addition, it was ensured that the participants only wore their school uniforms during this process. For height measurements, the participants were requested to stand upright with no footwear on a flat surface with their back of the heels and occiput against the equipment. The weight and height were recorded to the precision of 0.1 kg and 0.1 cm, respectively. For waist circumference, measurements were taken using a plastic nonelastic tape placed at the midpoint of the last rib and the top of the hip bone (iliac crest).

The body mass index (BMI) was computed using the height and weight data provided by dividing the participant's weight (kg) by the squared height (m^2^). The indices of waist-to-height ratio (WHtR) were calculated based on the ratio of the waist circumferences (WC) (cm) to height (cm). Conicity index (CI), an anthropometric measurement that is used to assess central adiposity, is calculated using

(2) $$\begin{array}{c} \text{Conicity index }\left(\text{CI}\right)=\frac{\text{waist circumference }\left(\text{m}\right)}{0.109\times \sqrt{\text{body weight }\left(\text{kg}\right)/\text{height}\left(\text{m}\right)}}. \end{array}$$

A digital blood pressure monitor was used for blood pressure measurements. The participants were requested to rest for 5 minutes to ensure that there was no exercise before the measurement. In addition, the participants were also checked to ensure that they did not consume any caffeine or medication before the measurement. For each participant, two measurements were taken. There was an interval of one minute between these two measurements. If the differences between these two readings were more than 5 mm Hg, a third reading would be taken. A third reading would also be taken when a participant was found to be prehypertension or hypertension. The average of these readings would be calculated as the final blood pressure reading for each of the participants. The participants were categorized into prehypertension, hypertension, and normal following the 4^th^ report on the diagnosis, evaluation, and treatment of high blood pressure in children and adolescents [40] where the cut-off point was based on age, sex, and height.

A total of 2461 sample data with a slightly higher number of females (*n* = 1428, 58%) compared to the number of males (*n* = 1033, 42%) was collected. This sample size is higher than the needed minimum sample size determined using Equation (1) and hence represents the Sarawak adolescent population. The mean age of the participants was 14.5 ± 1.50 years. In terms of ethnicity, the participants were mostly Iban, followed by Malay, Chinese, Bidayuh, and other ethnicities. The sociodemographic data included the age, sex, location, ethnicity, and whether the parent(s) was/were hypertensive of the participants, which is shown in Table 1. Most of the participants were from rural areas (74.2%). Referring to Table 2, the males had higher mean weight, height, and waist circumferences (WC), whereas the females showed higher mean body mass index (BMI) and waist-to-height ratio (WHtR). Both sexes exhibited the same mean *C* index. In terms of hypersensitivity (Table 3), it was found that more males were in the prehypertension and hypertension categories comparing to the females.

### 3.2. Methodology Design and Implementation

**Figure 1:**
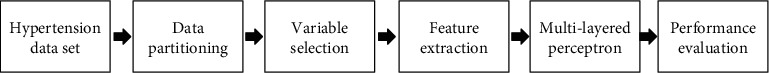
Overall classification procedures implemented in this study.

In this study, a multilayer perceptron feedforward neural network was designed and developed in the SAS Visual Data Mining and Machine Learning (VDMML) environment. The overall process of this classification procedure is shown in Figure 1. The detail of each step is presented below.

### 3.3. Data Partitioning

The statistical properties of the training, validation, and testing data play a vital role in ANN prediction and classification. The data set is partitioned into three subsets: 60% training, 30% validation, and 10% testing. For the original data set, the prehypertensive and hypertensive categories are grouped as one, resulted in binary output variables (normal and hypertensive) [32]. With this grouping, the total numbers of hypertensive and normal categories are 741 (30.1%) and 1720 (69.9%), respectively. Stratified random sampling according to the hypertensive and normal group ratio was done for the training, validation, and testing data sets. The distribution of the data obtained is shown in Table 4.

### 3.4. Variable Selection

In SAS VDDML environment, using the Fast Supervised Selection method, a set of input variables that mutually explain the maximum amount of variance contained in the target variable is chosen. The Fast Supervised Selection approach, which utilizes the Bayesian Information Criterion, penalises larger models more strongly and favours smaller models as a way of completing the selection process. With the cumulative variance cut-off set to 1.0, the Fast Supervised Selection process ends when the selected variables can explain this proportion of the overall variation.  Table 5 shows the proportion of the variance explained by these five selected parameters. From the total 11 input variables (sex, ethnicity, location, parents' hypertension history, age, weight, height, BMI, WC, WHtR, and C index), 5 parameters are selected: age, sex, ethnicity, weight, and WHtR.

### 3.5. Feature Extraction

In this part of the procedure, new feature(s) would be produced using the five variables obtained from the previous variable selection stage. The newly created features would capture the central characteristics of the selected data set and represent this data set in a lower-dimensional space. Principal Component Analysis (PCA) is a simple and most popular nonparametric method of obtaining the most relevant information from redundant or noisy data [41], and the new set features are called Principal Components (PCs). Using the PCA procedure, the features weight and WHtR variables are combined as a new variable, named Principal Component 1 (PC1). The use of PCA for feature extraction to reduce the feature dimension is well documented for clinical studies utilizing electronic healthcare records in [42]. The body weight is significantly correlated to the waist circumference [43]. The WHtR holds additional information on the height. Using the PCA process, a new feature (PC1) that captured the essential features from these two variables was created. Therefore, the final input features are reduced from five to four.

### 3.6. Artificial Neural Network Model

**Figure 2:**
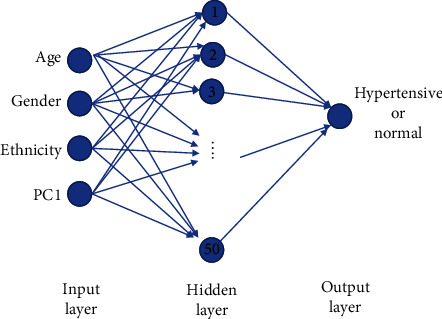
Multilayer perceptron model developed in this research study.

A multilayer perceptron neural network model with four input features, a single hidden layer of 50 hidden neurons, and one output layer is developed for the classification of hypertension and normal targets. The trial-and-error approach, which is a commonly utilized method [44], was applied in this study to determine the hidden neurons in the neural network model. Single-layer feedforward neural network possesses the universal approximation property [45].  Figure 2 shows the network architecture of the developed model.

The input variables are normalized using the *z*-score normalization method. The model properties are summarized in Table 6. Early stopping with five stagnations is carried out to avoid overtraining and to reduce training time. The model uses the Limited-Memory Broyden Fletcher Goldfarb Shanno (LBFGS), one of the quasi-Newton methods, that requires less computer memory.

## 4. Performance Evaluation

In this study, a few performance evaluation metrics are calculated to assess the performance of the developed multilayer perceptron model on hypertensive and normal patient classification.

In general, the performance of the binary classifier is grounded on the calculation of the following four parameters:

- True positive (TP) is defined as the number of hypertensive adolescents who are classified as hypertensive
- False negative (FN) is defined as the number of hypertensive adolescents who are classified as normal
- False positive (FP) is defined as the number of normal adolescents who are classified as hypertensive
- True negative (TN) is defined as the number of normal adolescents who are classified as normal

Using these four parameters, the sensitivity, specificity, precision, *F*-score, accuracy, misclassification rate, Receiver Operating Characteristic (ROC) Curve, and Area Under the ROC Curve (AUC) are calculated.

### 4.1. Bayes' Theorem

The sensitivity and specificity of a classifier can be used to assess its validity. However, these two performance indicators do not accurately reflect how well the model performs for a certain population given the incidence of a specific condition. In order to evaluate how relevant or therapeutically beneficial a test could be for a population, we need underlying information about the predicted incidence or prevalence of a disease. Bayes' Theorem is useful to explain this [46]. The formula of Bayes' Theorem is

(3) $$\begin{array}{c} P\left(A\;|\;B\right)=\frac{P\left(A\right)\times P\left(B\;|\;A\right)}{P\left(B\right)}, \end{array}$$

where *P*(*A*) is the unconditional probability of the disease in the population, i.e., prevalence; *P*(*B*) is the unconditional probability of the classifier/test returning positive; *P*(*B* | *A*) denotes the chances of event *B* given that event *A* occurring; and *P*(*A* | *B*) is the posterior probability which denotes the chance of *A* happening given *B*.

## 5. Results

### 5.1. Multilayer Perceptron Model Performance

**Figure 3:**
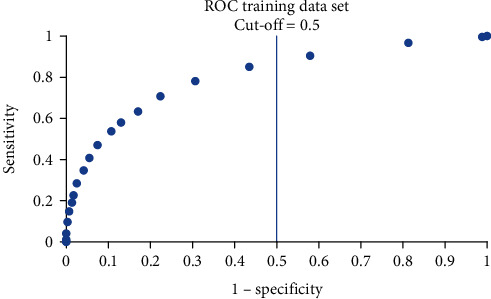
ROC of the training data set.

**Figure 4:**
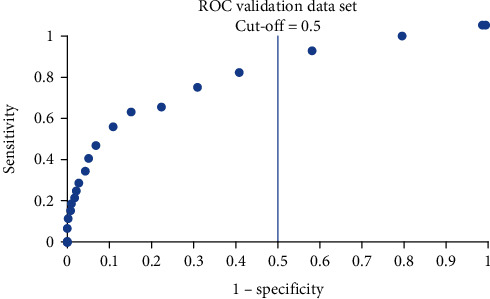
ROC of the validation data.

**Figure 5:**
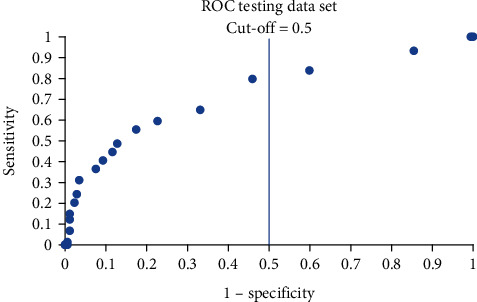
ROC of the testing data.

The distribution of the actual classification results for training, validation, and testing data sets are presented using the confusion matrix in Tables 7–9. Using the confusion matrix, the performance metrics of the developed multilayer perceptron model are presented in Table 10. From this table, it can be seen that the developed model managed to achieve a classification accuracy of 76% with 65% precision. The sensitivity and the specificity of the model are 0.41 and 0.91, respectively, while the AUC is 0.75. It should be noted that the cut-off point used for all these matrices is 0.5. The ROC for the training, validation, and testing data sets are shown in Figures 3, 4, and 5. The similar shape of the ROC in these figures indicates that the multilayer perceptron model did not overfit the data during training; i.e., the model demonstrated comparable predictive capability in the training, validation, and testing data sets. In other words, the developed model is well-generalized. This aligns with the similar sensitivity and specificity values achieved for these three sets of data, as shown in Table 10.

### 5.2. Variable Importance

A classification tree model is used to determine the variable importance in predicting the output variable. This is done in two steps. During the first step, the variable importance of each variable is calculated based on the change of Residual Sum of Square (RSS) when a split is found at a node. The maximum variable importance value is found from these values. In the second step, the relative variable importance value for each variable is calculated by dividing the variable importance by the maximum variable importance value. The detailed calculation of RSS could be found in [47].  Table 11 shows the variable importance and relative variable importance values of the four extracted features in this study. The classification results obtained without the feature extraction process in the multilayer perceptron neural network model developed in this study are included in Supplementary 1.

### 5.3. Reliability Test Using Bayes' Theorem

According to a study in year 2018, hypertension of secondary students in Sarawak was 30.1% [18]. The population of adolescents in Sarawak was 200130. Use Bayes' Theorem formula in Equation (3):

Event *A* denotes the prevalence of adolescent hypertension in Sarawak: *P*(*A*) = 0.301.

Use the sensitivity and specificity of the model developed in this study: sensitivity = 0.41 and specificity = 0.91.

Event *B* denotes the unconditional probability that our test coming up positive, which would include both true positive and false positive using our test. To calculate the total true positive (TTP) of our test (*N*_TP_),

(4) $$\begin{array}{c} N_{\text{TP}}=\text{hypertension prevalence}\times \text{total population}\times \text{sensitivity}=0.301\times 200130\times 0.41=24698. \end{array}$$

In order to calculate the total false positive (TFP) (*N*_FP_),

(5) $$\begin{array}{c} N_{\text{FP}}=\text{probability of not having hypertension}\times \text{total population}\times \left(1-\text{specificity}\right)=\left(1-0.301\right)\times 200130\times \left(1-0.91\right)=12590.\\ \text{Therefore},\text{the total positive }\left(\text{TP}\right)\text{ from our test}=N_{\text{TP}}+N_{\text{FP}}=24698+12590=37288. \end{array}$$

With this, *P*(*B*) = 37288/200130 = 0.1863.

From Equation (3):

(6) $$\begin{array}{c} P\left(A\;|\;B\right)=\frac{P\left(A\right)\times P\left(B\;|\;A\right)}{P\left(B\right)}. \end{array}$$

*P*(*A* | *B*) denotes the likelihood that an adolescent will have hypertension if our model indicates that he or she is hypertensive. *P*(*B* | *A*) defines the likelihood of receiving a positive result, regardless of whether it is a true-positive or false-positive. As a result, *P*(*B* | *A*) represents our sensitivity.

(7) $$\begin{array}{c} P\left(A\;|\;B\right)=\frac{0.301\times 0.41\;}{0.1863}=0.662=66.2\%. \end{array}$$

This indicates that an adolescent diagnosed with hypertension using our method has a 66.2% likelihood of being hypertensive.

## 6. Discussion

In this paper, a single hidden layer multilayer perceptron neural network was developed to model the hypertension classification problem in adolescents in Sarawak, Malaysia. The study manages to prove the claim that a multilayer neural network with one single hidden layer can model a broad range of challenges in the clinical domain.

A comparison of the performance of the developed model with the above-mentioned research is presented in Table 12. From the performance metrics obtained, it could be seen that the classification capability of the developed model (AUC = 0.75) is compatible with the use of deep learning for hypertension classification by López-Martínez et al. [27] (AUC = 0.77). Our model performs slightly better for all the other performance metrics. Besides the model developed by Bani-Salameh et al. [26] that did not report on the model's specificity, the other models, including the model developed in this research work, are better in terms of the models' specificity than the sensitivity. In other words, all these models are better at classifying normal patients than correctly classifying hypertensive patients. This could be the result of the imbalance data set used, which is higher in percentage of occurrence of normal than hypertensive patients.

Comparing to the model architecture developed by López-Martínez et al. [27], our network architecture is smaller (3 layers of 64 nodes, 32 nodes and 16 nodes, respectively, vs. single layer of 50 nodes). In addition, the percentage of normal (69.71%) and hypertensive patients (30.29%) used in [27] is similar to the ratio used in our study (30.1% normal and 69.9% hypertensive).

Another significant contribution of this research work is that only simple anthropometric measurements and sociodemographic data were collected during the cross-sectional study, i.e., age, sex, ethnicity, location, parent(s) hypertension history, weight, height, waist circumferences, and blood pressure. The variable selection process in the methodology in this study had selected age, sex, ethnicity, weight, and WHtR parameters as the input for the multilayer perceptron model. All the other research works included personal medical history data and lifestyle parameters. For example, smoking and kidney conditions were required in [27]; family history, history of hyperlipidemia, and coronary artery bypass graft in [29]; and diabetes data in [26, 27, 29]. Yet, self-reported diabetes and other medical history conditions are not reliable [36], and the lifestyle parameters reporting are subjective [37].

The analysis on the variable importance reveals that PC1, which is a new feature transformed from the weight and WHtR variable, is the most important feature for the classification of hypertensive and normal patients, followed by sex, age, and ethnicity. The use of ANN with these simple anthropometric measurements and sociodemographic data demonstrates the potential of the usage of the simple measurements for hypertension detection. However, as the predictive powers of anthropometric measures for hypertension are countries and ethnicities dependent [14], further studies on the use of these parameters on other geographical locations would better validate the usefulness of these inputs.

From the performance metrics presented for the training, validation, and testing data sets in Table 10, it could be concluded that the developed model is well-generalized. That is, the model can handle the unseen data. This is proved by the almost equal values obtained for the performance metrics of the training, validation, and testing data sets. This property is important in ensuring the usefulness of the model in real-life situation.

In terms of reliability, focusing whether the developed classifier is sufficiently trustworthy to be used in a clinical context, the prevalence of hypertension in a particular population should be taken into consideration. While a highly accurate classifier may be beneficial in populations with a greater prevalence of hypertension, it would be less instructive in populations with lower hypertension rates. In our work, if an adolescent is diagnosed with hypertension using our model, he or she has a 66.2% likelihood of having hypertension. Using Bayes' Theorem, we further examine our model with different adolescent hypertension prevalences of 10% and 50% in Sarawak. The results are summarized in Table 13. With a lower prevalence (10%), the model only managed to conclude a 33.6% chance of an adolescent of having hypertension. For a higher prevalence of 50%, the model could better conclude (82.0%) an adolescent of having hypertension. When the sensitivity of the model is improved to 90% and the specificity remains at 91%, at 30.1% of hypertension in the Sarawak adolescents population, the model may yield an 81.2% likelihood of an adolescent having hypertension.

## 7. Conclusions

In this research work, a multilayer perceptron neural network with one hidden layer of 50 hidden neurons was developed. The proposed model incorporating the variable selection and feature extraction procedure managed to improve the classification accuracy of the hypertension classification problem focusing on adolescents in Sarawak, Malaysia. The primary contribution of this study effort is the smaller designed network architecture, consisting of three layers with five inputs at the input layer, one hidden layer of fifty hidden neurons, and one output layer, for improved classification accuracy utilizing simple anthropometric measures and sociodemographic data. Furthermore, we demonstrated that if an adolescent tests positive for hypertension, the established model can predict that he or she has a 66.2% likelihood of developing hypertension. This model, which combines basic and straightforward anthropometric measures with sociodemographic data, i.e. age, sex, ethnicity, weight, and WHtR, is clinically useful for Sarawak adolescents with a hypertension prevalence of 30.1%.

Although the performance of the developed model is encouraging, the model could not serve as a clinical decision-making tool for diagnosing hypertensive patients. Nevertheless, the classification result could function as an early warning mechanism to alert patients on the possibility of being hypertensive.

The process to develop a multilayer perceptron neural network for adolescent hypertension classification is outlined clearly in this work. The knowledge gained in designing, developing, implementing, testing, and analyzing the network model is valuable in a future work to build an early warning tool for hypertension prediction. Such an early warning tool could serve as a cheap, simple, and rapid screening mechanism in helping the public on identifying the risk of hypertension, especially in settings when blood pressure monitoring equipment is not available. The developed model could only predict a 66.2% likelihood of an adolescent having hypertension, which is insufficient for the model to be utilized as a clinical decision-making tool. As a result, further research into the utilization of anthropometric data for hypertension prediction using machine learning algorithms is necessary. Furthermore, it would be necessary to assess whether additional training data will enhance the accuracy of the constructed model. This might be accomplished by data augmentation to generate additional data or through data collection.

## Figures and Tables

**(a):** 

	*n*	%
*Sex*		
Male (M)	1033	42.0
Female (F)	1428	58.0
*Ethnicity*		
Iban	737	29.9
Malay	681	27.7
Chinese	475	19.3
Bidayuh	256	10.4
Other	312	12.7
*Location*		
Urban	634	25.8
Rural	1827	74.2
*Parents hypertension history*		
One of the parents	448	18.2
Both parents	80	3.3
No	1933	78.5

**(b):** 

Age	Min	Max	Mean	Standard deviation
Male (M)	12	17	14.4	1.48
Female (F)	12	17	14.5	1.51

**Table 2:** Anthropometric data of the participants.

	Male (*n* = 1033)				Female (*n* = 1428)			
	Min	Max	Mean	Std	Min	Max	Mean	Std
Weight (kg)	24.4	121.8	55.5	14.78	21.2	109.4	51.0	12.80
Height (m)	1.3	1.8	1.6	0.08	1.24	1.78	1.5	0.06
BMI (kg/m^2^)	13.3	43.1	21.3	4.72	13.1	43.5	21.6	4.78
WC (cm)	51.5	125.0	71.3	11.56	50.0	655.0	70.2	18.46
WHtR	0.3	0.7	0.4	0.07	0.3	4.1	0.5	0.11
*C* index	0.8	1.4	1.1	0.07	0.8	11.0	1.1	0.27

**Table 3:** Blood pressure profile of the participants.

Sex	Male (*n* = 1033)		Female (*n* = 1428)	
Blood pressure	*n*	%	*n*	%
Prehypertension	199	19.3	125	8.8
Hypertension	232	22.5	185	13.0
Normal	602	58.3	1118	78.3

**Table 4:** Distribution of data using a stratified sampling method according to the ratio of hypertensive and normal groups.

Partition	Normal		Hypertensive		Total (*N* = 2461)	
	*n*	%	*n*	%	*n*	%
Training	1032	69.9	445	30.1	1477	60.0
Validation	516	69.9	222	30.1	738	30.0
Testing	172	69.9	74	30.1	246	10.0

**Table 5:** Proportion of variance explained for the five selected parameters through Fast Supervised Selection method.

Parameter	Proportion of variance explained
Weight	0.2314
Sex	0.2540
Ethnic	0.2614
WHtR	0.2657
Age	0.2682

**Table 6:** Multilayer perceptron parameter settings.

Parameter	Value
Input dimension	4
Number of output classes	2
Number of hidden layers	1
Hidden layer dimension	50
Hidden layer activation function	tanh
Momentum	0
Learning rate	0.0010
Optimization method	LBFGS (Limited-Memory Broyden Fletcher Goldfarb Shanno)

**Table 7:** Confusion matrix obtained using training data.

	Actual	
	Hypertensive	Normal
Prediction		
Hypertensive	204	66
Normal	241	966

**Table 8:** Confusion matrix obtained using validation data.

	Actual	
	Hypertensive	Normal
Prediction		
Hypertensive	99	44
Normal	123	472

**Table 9:** Confusion matrix obtained using testing data.

	Actual	
	Hypertensive	Normal
Prediction		
Hypertensive	30	16
Normal	44	156

**Table 10:** Classification results obtained for training, validation, and testing data sets.

Performance metrics	Training	Validation	Testing
Sensitivity	0.46	0.45	0.41
Specificity	0.94	0.91	0.91
Precision	0.76	0.69	0.65
F-score	0.57	0.54	0.50
Accuracy	0.79	0.77	0.76
Misclassification rate	0.21	0.23	0.24
AUC	0.82	0.79	0.75

**Table 11:** Variable importance.

Variable	Variable importance	Relative variable importance
PC1	246.67	1.00
Sex	37.88	0.15
Age	33.02	0.13
Ethnicity	32.44	0.13

**Table 12:** Performance metrics comparison.

Our model	Sensitivity	Specificity	Precision	*F*-score	Accuracy	AUC
	0.41	0.91	0.65	0.50	0.76	0.75
López-Martínez et al. [27]	0.40	0.87	0.58	0.47	0.73	0.77
Bani-Salameh et al. [26]	0.69	—	0.68	0.68	0.68	0.62
Sakr et al. [29]	0.31	0.88	0.57	0.39	—	0.67

**Table 13:** Model reliability testing using Bayes' Theorem for different prevalence and sensitivity levels. The model reliability on current hypertension prevalence in Sarawak adolescents is highlighted.

Prevalence	Sensitivity	Specificity	TTP	TFP	TP	*P*(*B*)	*P*(*A* | *B*)
10%	41%	91%	8205	16210	24415	0.1220	33.6%
50%	41%	91%	41026	9005	50031	0.2500	82.0%
30.1%	65%	91%	39155	12590	51745	0.2586	75.7%
30.1%	90%	91%	54215	12590	66805	0.3338	81.2%
30.1%	41%	91%	24698	12590	37288	0.1863	66.2%

## Data Availability

The data underlying the results presented in the study are available upon request to the coauthor of the paper Dr. Cheah Whye Lian upon request by email (wlcheah@unimas.my).

## Supplementary Materials

Supplementary Materials A more detail description of the data used in this study could be found in [17, 18]. Supplementary 1: results obtained using the multilayer perceptron model without feature extraction process.
