# Supplementary material for: A Multilayer Perceptron Neural Network Model to Classify Hypertension in Adolescents Using Anthropometric Measurements: A Cross-Sectional Study in Sarawak, Malaysia
Source: Comput Math Methods Med. 2021 Dec 7;2021:2794888. doi: 10.1155/2021/2794888 (PMC8670914; doi:10.1155/2021/2794888)
Supplement: Supplementary Materials — A more detail description of the data used in this study could be found in [17, 18]. Supplementary 1: results obtained using the multilayer perceptron model without feature extraction process. [file 2794888.f1.docx]

**Supplementary 1** – Results obtained using the multi-layer perceptron model without feature extraction process

Table A1. Training data

|  | | Actual | |
| --- | --- | --- | --- |
|  |  | Hypertensive | Normal |
| Prediction | Hypertensive | 207 | 78 |
|  | Normal | 238 | 954 |

Table A2. Validation data

|  | | Actual | |
| --- | --- | --- | --- |
|  |  | Hypertensive | Normal |
| Prediction | Hypertensive | 76 | 25 |
|  | Normal | 146 | 491 |

Table A3. Testing data

|  | | Actual | |
| --- | --- | --- | --- |
|  |  | Hypertensive | Normal |
| Prediction | Hypertensive | 29 | 17 |
|  | Normal | 45 | 155 |

Table A4. Classification results obtained for training, validation and testing data sets.

| **Performance Metrics** | **Training** | **Validation** | **Testing** |
| --- | --- | --- | --- |
| Sensitivity | 0.47 | 0.34 | 0.39 |
| Specificity | 0.92 | 0.95 | 0.90 |
| Precision | 0.73 | 0.75 | 0.63 |
| F-score | 0.57 | 0.47 | 0.48 |
| Accuracy | 0.79 | 0.77 | 0.75 |
| Misclassification Rate | 0.21 | 0.23 | 0.25 |
| AUC | 0.82 | 0.80 | 0.76 |

According to Bayes’ Theorem, with 30.1% hypertension prevalence in the Sarawak adolescents population,

$$P\left( A | B \right)=\frac{0.301 \times0.39}{0.1873}=0.627=62.7\%$$
